# Supplementary material for: Evaluation of Antibodies Induced by Melanoma Helper Peptide Vaccine and Their Modulation by Vaccine Adjuvants
Source: Vaccines (Basel). 2026 Feb 21;14(2):195. doi: 10.3390/vaccines14020195 (PMC12944968; doi:10.3390/vaccines14020195)
Supplement: Supplementary file 1 [file vaccines-14-00195-s001.zip › Supplemental Material Table S1 6MHP Sequences.pdf]

Table S1. Peptide sequences from 6MHP Vaccine

Allele	Sequence	Epitope
HLA-DR4	AQNILLSNAPLGPQFP	$Tyrosinase_{56-70}$
HLA-DR15	FLLHHAFVDSIFEQWLQRHRP	$Tyrosinase_{386-406}$
HLA-DR4	RNGYRALMDKSLHVGTQCALTRR	Melan-A/MART-1_{51-73}
HLA-DR11	TSYVKVLHHMVKISG	MAGE-$3_{281-295}$
HLA-DR13	LLKYRAREPVTKAE	MAGE-1,2,3,$6_{121-134}$
HLA-DR1 & HLA-DR4	WNRQLYPEWTEAQRLD	$gp100_{44-59}$
