# Supplementary material for: Evaluation of Antibodies Induced by Melanoma Helper Peptide Vaccine and Their Modulation by Vaccine Adjuvants
Source: Vaccines (Basel). 2026 Feb 21;14(2):195. doi: 10.3390/vaccines14020195 (PMC12944968; doi:10.3390/vaccines14020195)
Supplement: Supplementary file 1 [file vaccines-14-00195-s001.zip › Supplemental Material Table S2 Mel41 Patient Samples Used.pdf]

Table S2. Mel41 Serum Samples Included in Study

Patient #	VMM	Trial Arm	Serum used (weeks post-vaccination)
8	VMM 871	B	Week 12
7	VMM 729	C	Week 12
6	VMM 719	C	Week 12
5	VMM 701	A	Week 12
4	VMM 699	B	Week 12
3	VMM 683	C	Week 12
2	VMM 625	C	Week 12
1	VMM 485	C	Week 12
