# Supplementary material for: Evaluation of Antibodies Induced by Melanoma Helper Peptide Vaccine and Their Modulation by Vaccine Adjuvants
Source: Vaccines (Basel). 2026 Feb 21;14(2):195. doi: 10.3390/vaccines14020195 (PMC12944968; doi:10.3390/vaccines14020195)
Supplement: Supplementary file 1 [file vaccines-14-00195-s001.zip › Supplemental Material Table S3 Mel63 Samples and Antibody Concentrations.pdf]

Table S3. Mel63 Patients Included in Study and IgG Concentrations (μg/mL)

Patient	Trial Arm	Serum used (weeks post-vaccination)	IgG1	IgG2	IgG3	IgG4	IgG Total
VMM1221	A	Week 18	0.32	0.96	6.8	0	16
VMM1223	A	Week 18	0.28	0.96	6.3	0	8.0
VMM1225	B	Week 18	0.31	0	19	0	3.4
VMM1227	B	Week 18	18	1.8	12	0	33
VMM1233	B	Week 18	0.28	0	1.4	0	3.8
VMM1234	B	Week 18	0.89	0	3.6	0	6.5
VMM1246	B	Week 18	3.3	1.1	4.5	0	7.6
VMM1226	C	Week 26	3.5	1.6	22	0	49
VMM1229	C	Week 26	35	1.5	54	0	90†
VMM1239	C	Week 26	1.5	0.86	45	0	86
VMM1243	C	Week 18	1.7	1.2	13	0	31
VMM1248	C	Week 18	1.2	3.2	51	0	52
VMM1231	D	Week 26	3.3	1.1	82	0	87
VMM1232	D	Week 26	49	0	34	0	70
VMM1238	D	Week 26	79	1.2	5.5	0	76
VMM1240	D	Week 26	0.93	0	0	0	0.10
VMM1241	D	Week 18	1.8	0	0	0	0.05
VMM1242	D	Week 18	1.2	0	36	0	50
VMM1249	D	Week 26	8.0	1.5	17	0	30
VMM1254	D	Week 26	28	0	68	0.46	81
VMM1255	D	Week 18	17	1.1	44	0	82
VMM1262	D	Week 18	1.2	0.86	34	0	37
VMM1263	D	Week 12	0.98	1.1	16	0.48	42
VMM1264	D	Week 18	20	2.3	26	0.70	70
VMM1270	D	Week 12	2.1	2.5	20	0.77	40
VMM1277	D	Week 12	8.3	1.8	15	0.47	46
Number (%) positive by arm	A		0 (0%)	0 (0%)	2 (100%)	0 (0%)	2 (100%)
	B		2 (40%)	0 (0%)	4 (80%)	0 (0%)	5 (100%)
	C		2 (40%)	0 (0%)	5 (100%)	0 (0%)	5 (100%)
	D		8 (57%)	0 (0%)	12 (86%)	0 (0%)	12 (86%)
Overall			12 (46%)	0 (0%)	23 (88%)	0 (0%)	24 (92%)

*Shaded boxes are ones that did not meet the threshold of >10x the FU of normal donor serum to be considered a positive response

*Concentrations of 0 were below the detectable range

†IgG total was not properly detected for this patient, so the sum of IgG subclasses is used in place of total IgG value
